# Concentration dataset of 8 selected trace elements in cultured rainbow trout (*Oncorhynchus mykiss*) and dietary exposure risks in the Missouri adult population

**DOI:** 10.1016/j.dib.2021.107502

**Published:** 2021-10-23

**Authors:** Abua Ikem, Jimmie Garth, James Wetzel, Gabrielle Caldwell

**Affiliations:** a Department of Agriculture and Environmental Sciences, Lincoln University, Jefferson City, MO 65101, United States; b Cooperative Research Programs, Lincoln University, Jefferson City, MO 65101, United States

**Keywords:** Aquaculture, *Oncorhynchus mykiss*, Essential elements, Non-essential elements, Dietary intake, Health risk, Adult population, Missouri

## Abstract

Aquaculture contributes to the global animal protein supply and the prevention of malnutrition and diet-related diseases (FAO (Food and Agriculture Organization of the United Nations); Fiorella et al., 2021). In particular, fish is a significant source of animal protein, fatty acids (e.g., docosahexaenoic acid, eicosapentaenoic acid), vitamins, and essential trace elements in the human diet. Nonetheless, fish bioaccumulates metals from their diet and habitat. This data article includes information on the concentrations of 4 essential and 4 non-essential trace elements in cultured rainbow trout (*Oncorhynchus mykiss*) and the human health risks through fish consumption in the adult population. Concentrations of four essential (Cr, Cu, Ni, and Zn) and four non-essential (As, Cd, Pb, and Hg) elements were quantified in ninety-one *O. mykiss* samples from an in-door production system. Total mercury (THg) levels in samples were determined by AAS, and other analyzed trace elements were measured by ICP-OES after microwave-assisted acid digestion. The highest concentrations of metals/metalloid (mg/kg wet weight) in fish muscle were Cr (0.44), Cu (4.21), Ni (1.01), As (0.47), Cd (0.045), Pb (0.65), THg (0.029), and Zn (6.21). The average concentrations of Cr and Pb exceeded their respective maximum limit. In most cases, median metal concentrations significantly (*p* < 0.05) differed across the fish size groups (small: 264–295 mm; medium: 300–395 mm; and large: 400–552 mm). The median concentrations of Cd, Zn, and THg in the large size group differed significantly (*p* < 0.05) between genders. The estimated weekly intake values of quantified elements from muscle were below the provisional tolerable weekly intakes. Non-carcinogenic risk assessment in adult consumers, being below one (THQ ≤ 1; and TTHQ ≤ 1), indicated an insignificant health hazard. The estimated incremental and cumulative cancer risks in the adult class through Cr, As, Ni, and Pb exposure were greater than the benchmark (10^−5^). Accordingly, high dietary intake of metals/metalloid from cultured *O. mykiss* posed a risk of carcinogenesis in the adult risk class.

## Highlights

• Concentrations of eight trace elements in *O. mykiss* from aquaculture.
• Only Cr, in some samples, exceeded the permissible limit.
• EWI values of analyzed trace elements were below the PTWIs.
• Arsenic was the highest contributor to non-carcinogenic risk in adult consumers.
• Cancer risk of As, Cd, Cr, and Pb in the adult population is probable.
• High intake per week of *O. mykiss* posed health risks to the adult risk group.

## Specifications Table

**Table:** 

Subject	Environmental Science, Food Science, Public Health
Specific subject area	Environmental Chemistry, Food Toxicology, Exposure Assessment
Type of data	Tables and Figures Excel spreadsheet
How data were acquired	Wet chemistry involved sample pretreatment using the UltraWAVE™ microwave digestion (Milestone, Inc., CT, USA) followed by the analysis of 7 elements (As, Cd, Cr, Cu, Ni, Pb, and Zn) using ICP - OES (Agilent 5110 ICP–OES SVDV; Agilent Technologies, Inc., USA). Total mercury (THg) analysis was performed by AAS (DMA: Direct Mercury Analyzer; model DMA-80 *Evo* TRICELL, Milestone, Inc., USA).
Data format	Raw/Analyzed; Raw dataset is included in the article as a supplementary excel file (.xlxs). Analyzed data were compared with prescribed limits for fish and benchmarks for non-carcinogenic and carcinogenic risks in adults.
Parameters for data collection	Rainbow trout (*O. mykiss*) morphometric measurements (length and weight data) were recorded. Further, samples were grouped into three fish sizes (small: 264–295 mm); medium (300–395 mm); and large (400–552 mm in length). Trace elements (As, Cd, Cr, Cu, Ni, Pb, Zn, and THg) were determined in fish muscle samples (all samples; *n* = 91).
Description of data collection	*O. mykiss* were grown to harvest age (6 months) in an in-door production system. Fish samples were collected, and the physical measurements of fish fork length and fish weight were performed. Between 30 and 50 g of filleted fish were placed in coded Ziploc bags. All samples were frozen at -40 °C until analyses. *O. mykiss* was identified using the FishBase database [3]. Sample pretreatment was conducted in a single reaction chamber (SRC) UltraWAVE™ microwave digestion system (Milestone Inc., CT, USA). The process was as follows: 5.0 mL of HNO_3_ (65%, w/w; trace metal grade) was added to a known weight of a fish muscle (1.07 ± 0.03 g wet weight (ww)) in an acid-cleaned quartz digestion tube. The heating steps were: 1500 W: ramp 5 min to 70 °C; 1500 W: ramp 5 min to 100 °C; 1500 W: ramp 5 min to 180 °C; 1500 W: ramp 10 min to 250 °C; 1500 W: hold at 250 °C; and cooling and depressurization of the SRC. Concentrations of essential (Cr, Cu, Ni, and Zn) and non-essential (As, Cd, and Pb) in digested fish samples were quantified by ICP-OES. THg levels in fish muscle samples were determined by AAS. Dietary intakes of trace elements and the health indices in the adult population were calculated from the concentration dataset of eight elements (As, Cd, Cr, Cu, Ni, Pb, THg, and Zn), ingestion rate, body weight, oral reference dose (RfD_o_), and other variables.
Experimental features	THg in fish muscle was determined using the DMA and following the US EPA Method 7473 [4]. Other trace elements (As, Cd, Cr, Cu, Ni, Pb, and Zn) were measured using Agilent ICP – OES. Calculation of the daily/weekly intakes and non-carcinogenic and carcinogenic risks via fish consumption in the adult class was undertaken using concentration values of analyzed elements.
Data source location	Institution: Lincoln University aquaculture farm City/Town/Region: Jefferson City, Missouri. Country: United States. Latitude and longitude (and GPS coordinates, if possible) for collected samples/data: 38.527986^o^ and -92.137148^o^
Data accessibility	All the data are included in this article.

## Value of the Data

• Aquaculture is a significant component in the supply of fish, fishery products, and edible aquatic plants [1]. Fish is a significant source of high-quality proteins, vitamins, and essential nutrients in the diet of adults and children [2,5] but contaminants and their potential transfers to humans is a global problem. Thus, this dataset provides new information on the concentrations of eight trace elements in *O. mykiss* from aquaculture.
• This dataset describes the health risk indices [target hazard quotient (THQ), total THQ (TTHQ), incremental lifetime cancer risk (ILCR), and cumulative lifetime cancer risk (∑ILCR)] from intake of metals from cultured fish by the adult population. Metal levels in fish, as in this work, are important in addressing disease burdens in population risk groups (e.g., women, children, adults).
• This article highlights the nutritional and toxicological aspects of cultured *O. mykiss* and serves as baseline data in future studies.
• Furthermore, concentration datasets on metals/metalloid in feeds, cultured fish, and water quality are scarce. This data would expand our knowledge, benefit breeders, and aid aquaculture policymakers and industry actors towards improved aquaculture production regulations.

## 1 Data Description

### 1.1 Study area

**Fig. 1:**
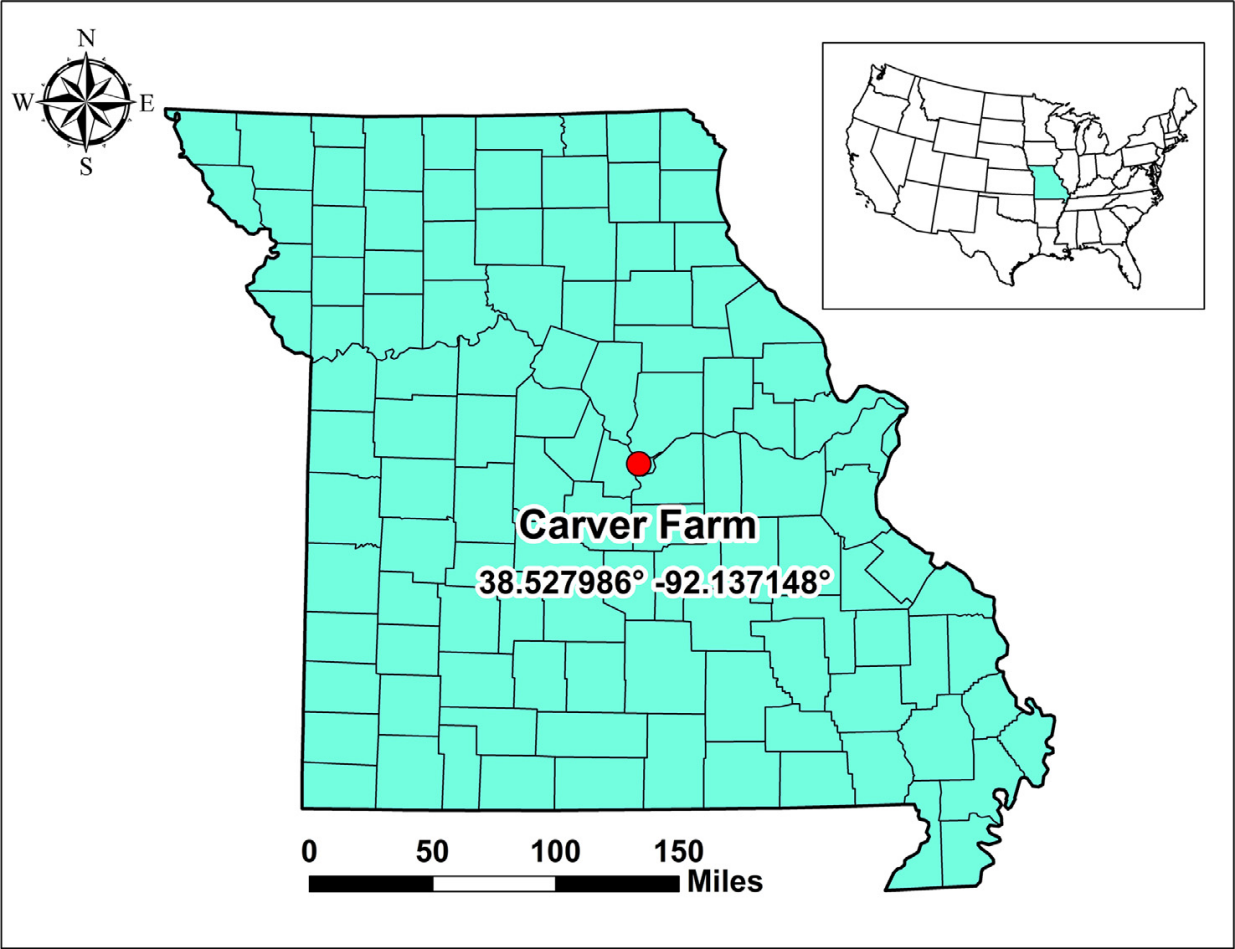
Location of the in-door aquaculture production site (Carver farm) in Missouri. (**Source**: [6]).

The location of the aquaculture production facility (Coordinates: 38.527986^o^, -92.137148^o^) is shown in Fig. 1. The fish production tanks were fed groundwater from a deep well near the facility.

### 1.2 Analytical data

**Fig. 2:**
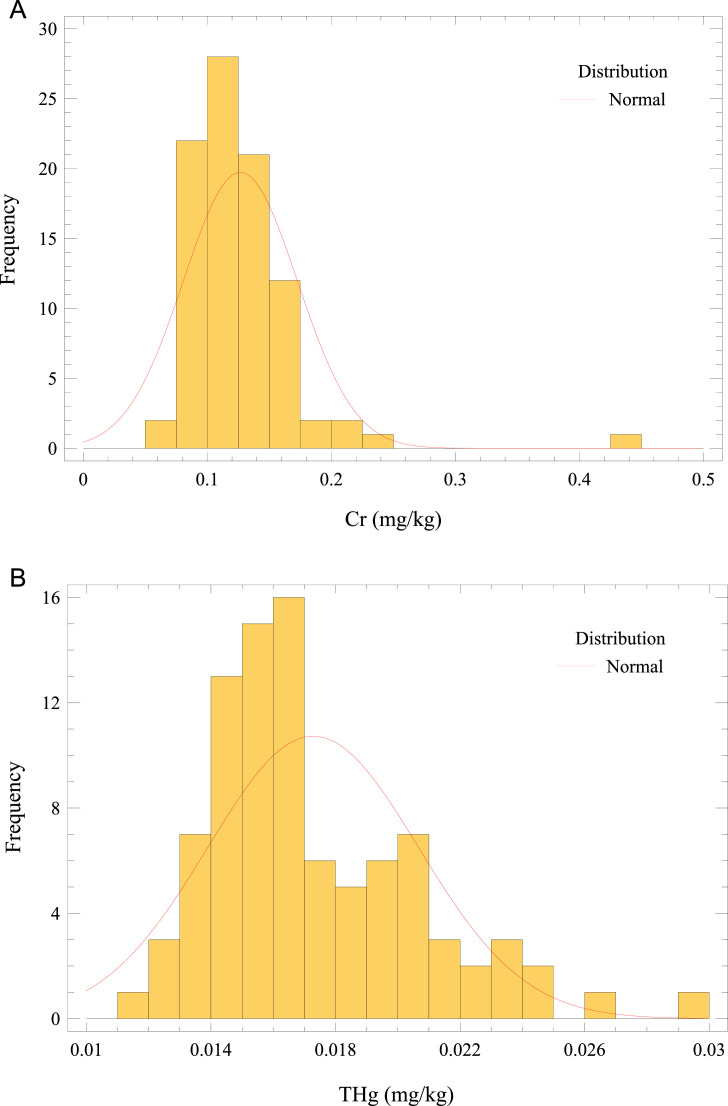

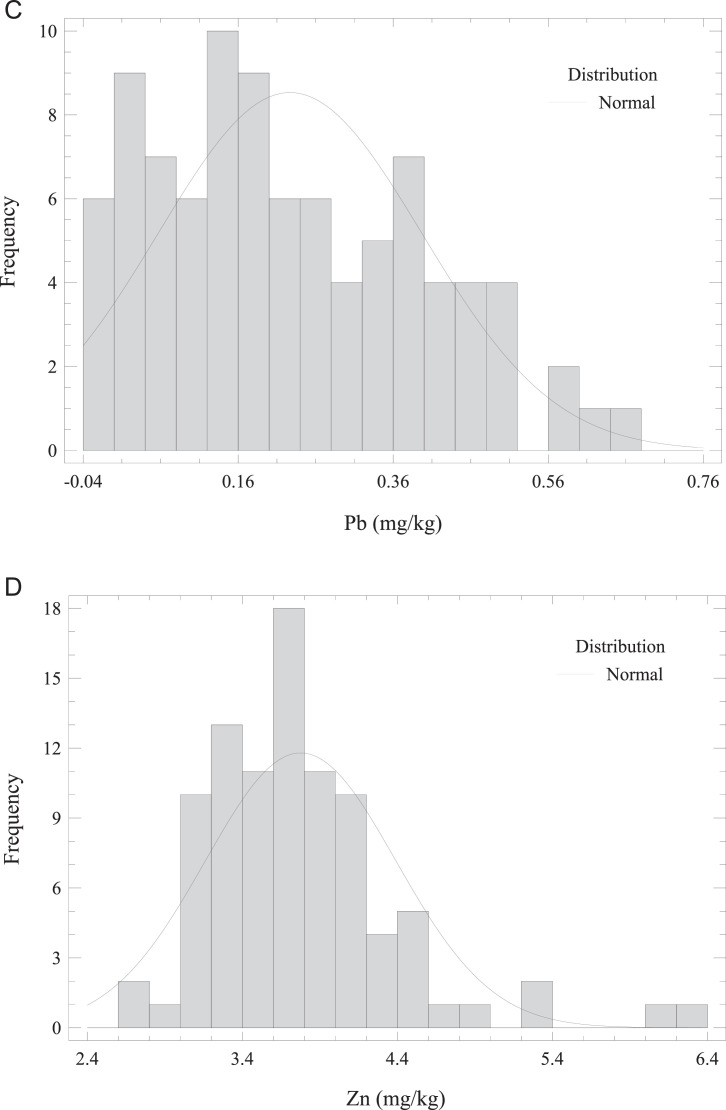

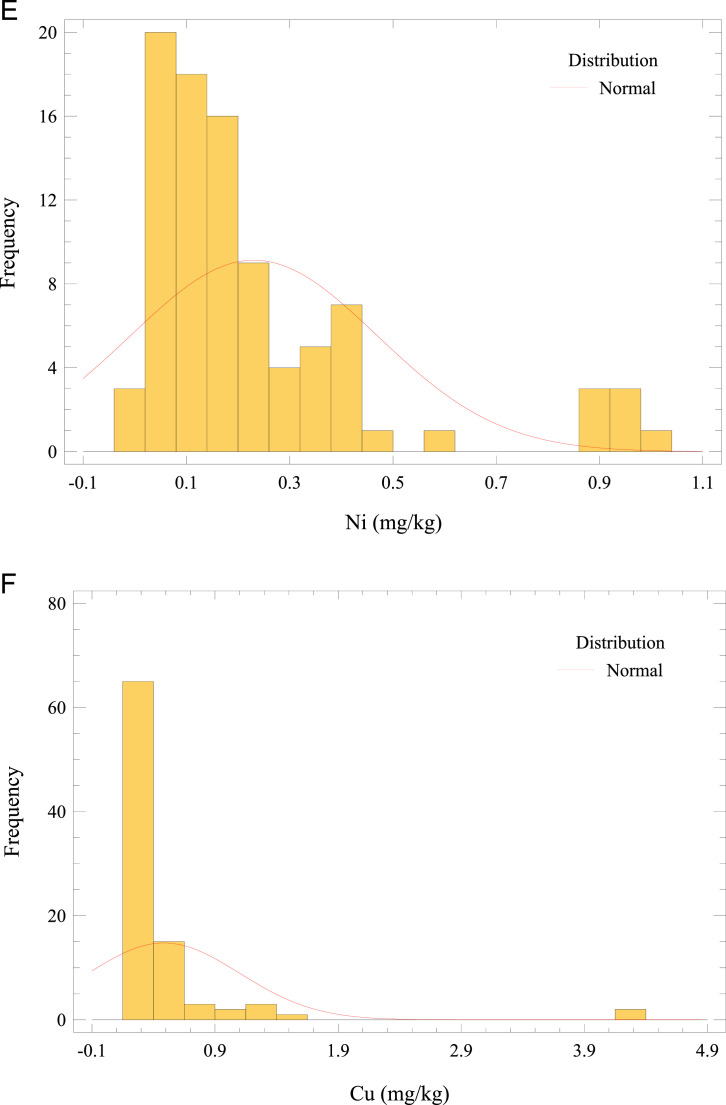

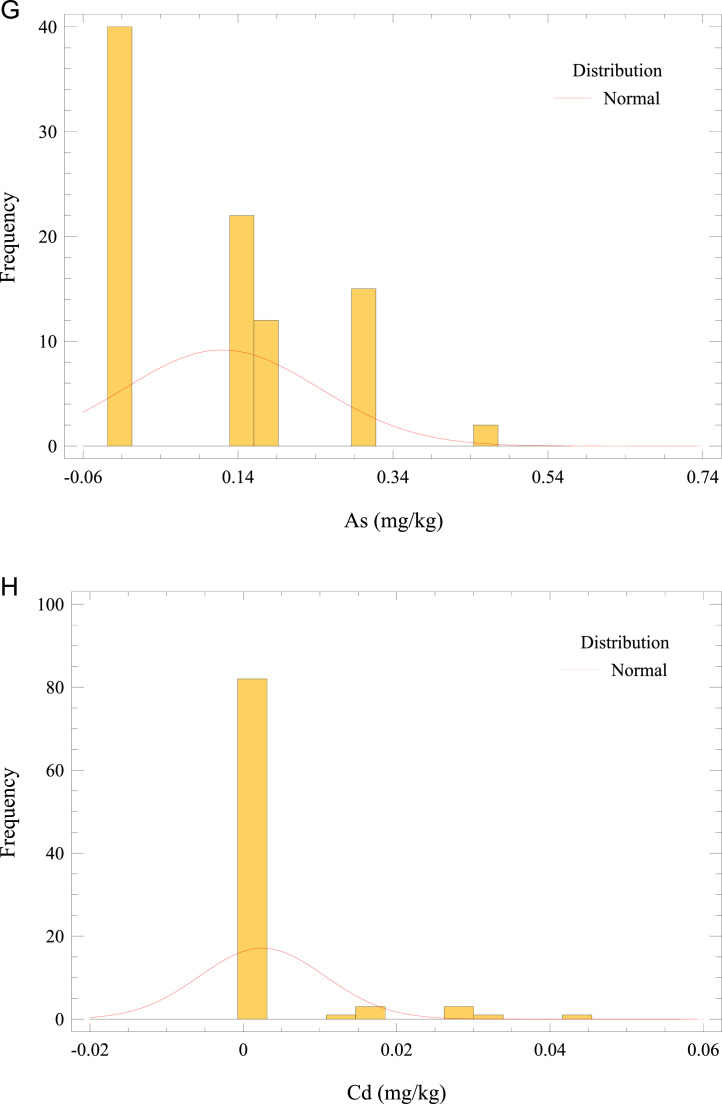
Frequency distribution of (a) Cr, (b) THg, (c) Pb, (d) Zn, (e) Ni, (f) Cu, (g) As, and (h) Cd concentrations in *O. mykiss* from aquaculture.

This article describes the concentration dataset of 4 essential (Cu, Cr, Ni, and Zn) and 4 non-essential trace elements (Pb, As, Cd, and THg) in *O. mykiss* reared in an in-door production system (Missouri). Besides, this paper provides estimates of metals/metalloid exposure from fish muscle consumption in the adult population. A Microsoft Excel worksheet of the raw data is reported as Supplementary Material. The raw data for trace elements in fish muscle is presented as the average of triplicate analyses of each sample. Furthermore, the Supplementary Material summarizes the elemental profiles of the feeds (pellet sizes categorized according to fish size or age), and the water quality constituents (deep well, inlet tank containing activated carbon adsorbent, and fish tank). Fig. 2 a–h presents the frequency distributions for analyzed elements in fish muscle. Regarding trace elements in fish muscle, the summary statistics (average, standard deviation, minimum and maximum values) and the comparison with maximum limits (MLs) [7 –10] are given in Table 1. The average Cr concentration in *O. mykiss* muscle exceeded the ML with 67% of samples above the threshold. Moreover, the levels of other elements were below the respective prescribed guideline.

**Table 1:** Mean elemental concentrations (± standard deviation; mg/kg ww; *n* = 91) in *O. mykiss* from aquaculture.

Summary statistics	Fish weight (g)	Fish length (mm)	As	Cd	Cr	Cu	Ni	Pb	Zn	THg
Average	714	355	0.12	0.002	0.13	0.48	0.23	0.23	3.78	0.017
SD	482	75	0.12	0.008	0.05	0.61	0.24	0.17	0.62	0.003
Count	91	91	91	91	91	91	91	91	91	91
SE	50.6	8.0	0.013	0.001	0.004	0.07	0.03	0.02	0.07	0.0004
Median	528	335	0.15	<LOD	0.12	0.32	0.14	0.19	3.70	0.017
Minimum	196	245	<LOD	<LOD	0.063	0.18	<LOD	<LOD	2.67	0.011
Maximum	2282	552	0.47	0.045	0.44	4.21	1.01	0.65	6.21	0.029
P50	528	335	0.15	<LOD	0.12	0.32	0.14	0.19	3.70	0.017
P90	1346	445	0.31	<LOD	0.16	0.68	0.44	0.47	4.41	0.022
P95	1591	474	0.32	0.022	0.18	1.32	0.90	0.51	4.80	0.024
Maximum limits			3.5 a	0.2 b 0.05 c	0.1 d	20 b	-	2.0 b 0.3 c	50 b 30 d	0.5 b
Exceedance (%)			0	0	67	0	-	0	0	0

SD is standard deviation; SE is the standard error; P50 is the 50th percentile; P90 is the 90th percentile; P95 is the 95th percentile; Age of all fish is 6 months

a [7]

b [8]

c [9]

d [10].

**Fig. 3:**
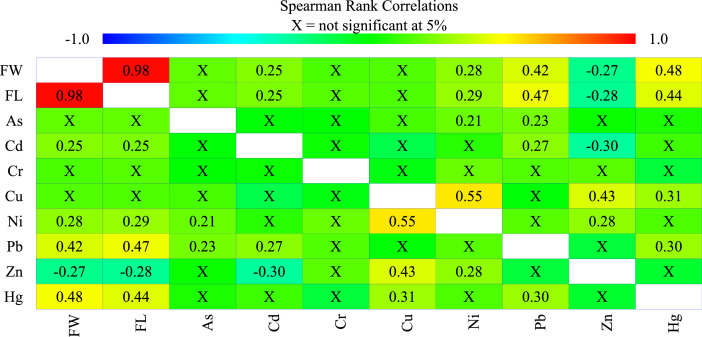
Spearman's rank coefficients (r) for correlation of variables measured in *O. mykiss* from aquaculture.

**Fig. 4:**
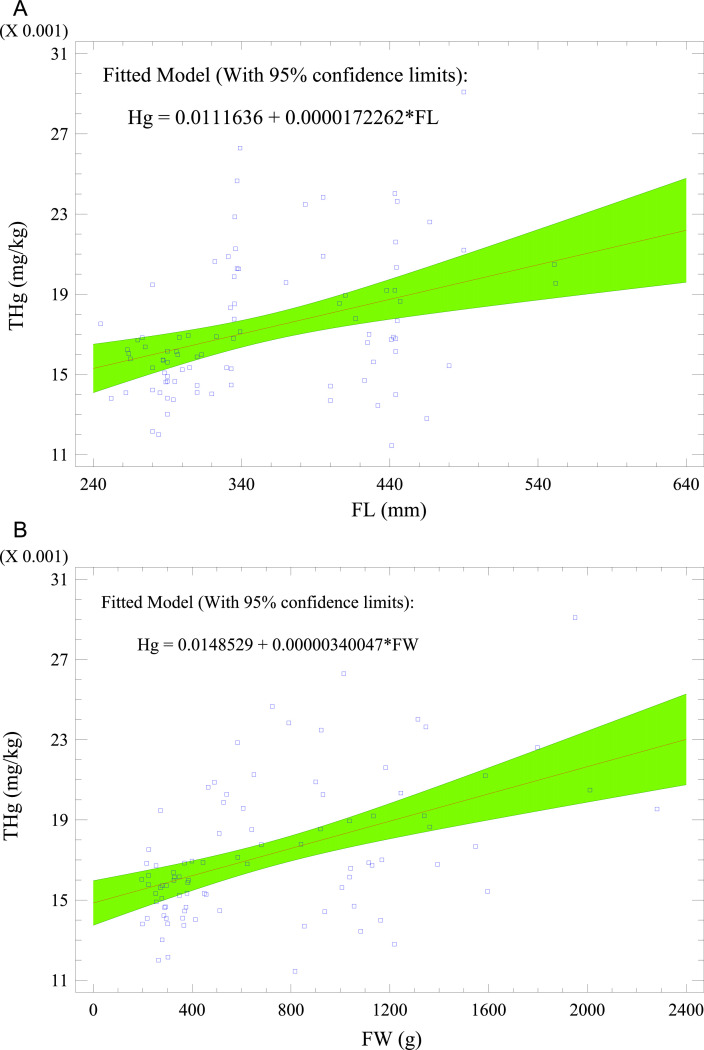

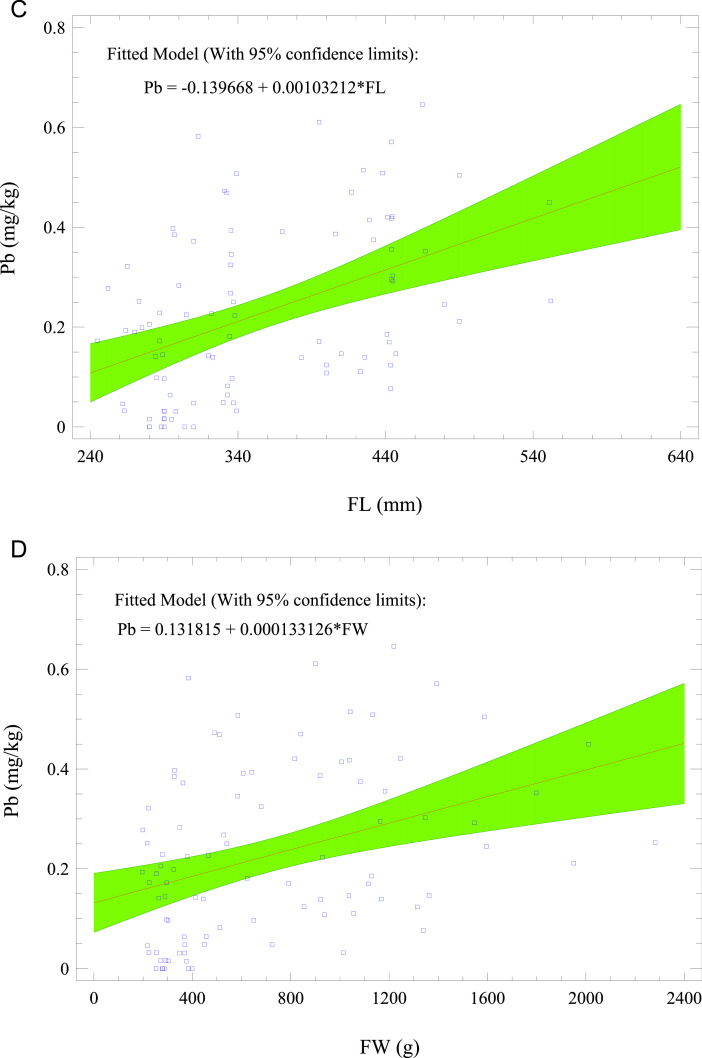
Fitted models (with 95% confidence limits) for the prediction of (a) THg with FL; (b) THg with FW; (c) Pb with FL; and (d) Pb with FW for cultured *O. mykiss*. FL = fish length, and FW = fish weight.

Fig. 3 shows Spearman's correlation coefficients of the variables. The growth factors [fish weight (FW) and fish length (FL)] correlated excellently (i.e., FW vs. FL: *r*^2^ = 0.98; *p* < 0.05). THg and Pb were assimilated during fish growth. Consequently, the growth factors (FW and FL) moderately correlated with THg and Pb (*r*^2^: 0.42 – 0.48; *p* < 0.05). Pb and THg, being bioaccumulative metals, were predicted using the morphometric values of the fish samples. Fig. 4 a–d presents the fitted models (with 95% confidence limits) for the prediction of THg and Pb with FL and FW. Other significant correlations were Zn vs. Cu (*r*^2^: 0.43; *p <* 0.05) and Cu vs. Ni (*r*^2^: 0.55; *p <* 0.05), which indicated their common origins.

Non-parametric Kruskal-Wallis comparison of median concentrations indicated significant differences in Cu (*p* = 0.0197412); As (*p =* 0.0155674); Cd (*p =* 0.01447); Ni (*p =* 0.000390239); Pb (*p =* 0.0000549923); and THg (*p =* 0.000128496) across the fish size groups (small: 264–295 mm; medium: 300–395 mm; and large: 400–552 mm). Withal, the median concentrations of Cd (*p =* 0.0288071); Zn (*p =* 0.0296971); and THg (*p =* 0.00638073) differed significantly between the sexes in the large fish size group (males: *n* = 16; females: *n* = 16).

Table 2 shows the input variables [5,11,12] for the estimation of daily/weekly (EDI/EWI) intakes of trace elements via fish consumption, EDI/EWI (Table 3), and the health indices for non-carcinogenic (Table 4), and carcinogenic (Table 5) human health risks in the Missouri adult population. Weekly intake estimates of the trace elements were below the provisional tolerable weekly intake values (PTWIs), which indicated no harm from the consumption of fish muscle by the adult risk class. Yet, the calculated non-carcinogenic risk was negligible and posed no hazard to adult consumers. Concerning cancer risk, the estimated cancer risk indices (ILCR > 10^−5^; and ∑ILCR > 10^−5^), indicated a probable risk of carcinogenesis from exposure to Cd, As, Cr, and Pb in the adult risk group. The observed cancer risk exceedances (above the benchmark: 10^−5^) in parenthesis for fish muscle samples were ILRC_As_ (69%); ILCR_Ni_ (98%); ILCR_Cr_ (100%); ILCR_Pb_ (75%); and ∑ILCR (100%). Herewith, moderate consumption of *O. mykiss* is recommended to safeguard public health.

**Table 2:** Input variables for the estimation of daily/weekly intakes in *O. mykiss* muscle, and the non-carcinogenic and carcinogenic risks in the adult population.

Input variable	Define	Unit	Value	Reference
C	Average element concentration	µg/g; ww	-	
IR	Daily ingestion rate	g/person-day	Adults: 32.5714	
F	Weekly ingestion rate	g/week	Adults: 228	
EFr	Exposure frequency	day/year	365	
ED	Exposure duration	year	Adults: 79	
RfD_o_	Oral reference dose	mg/kg-d	As (iAs; the most toxic form): 3.0 × 10^−4^, Cd: 1.0 × 10^−3^, Cu: 4.0 × 10^−2^, Cr (as Cr (VI)): 3.0 × 10^−3^, Pb: 3.6 × 10^−2^, Ni subsulfide: 1.1 × 10^−2^, Hg: 1.0 × 10^−4^, and Zn: 3.0 × 10^−1^	[5]
BW	Average body weight	kg	Adults: 70	[11]
AET	Averaging exposure time	day	Adults: 28835	
CSF	Cancer slope factor		As (inorganic arsenic): 1.5, Cr (VI): 0.5, Ni (nickel subsulfide): 1.7, and Pb (subacetate): 0.0085	[12]

**Table 3:** Mean estimated daily (± SD; µg/kg bw per day) and weekly (± SD; µg/kg bw per week; *n* = 91) intakes of metals/metalloid from *O. mykiss* muscle in comparison with guideline values (µg/kg body weight per week).

Element	Weekly intake guideline	EDI	EWI	% (EWI/Guideline)
As	15	0.06 ± 0.06	0.39 ± 0.41	2.6
Cd	7	0.001 ± 0.004	0.008 ± 0.027	0.1
Cr	21	0.06 ± 0.02	0.41 ± 0.15	2.0
Cu	3500	0.23 ± 0.29	1.58 ± 2.00	0.1
Ni	140	0.11 ± 0.11	0.75 ± 0.78	0.5
Pb	25	0.11 ± 0.08	0.74 ± 0.55	3.0
Zn	7000	1.76 ± 0.29	12.3 ± 2.00	0.2
THg	4	0.008 ± 0.002	0.056 ± 0.011	1.4

SD = Standard deviation; EDI = estimated daily intake; EWI = estimated weekly intake.

**Table 4:** Non-cancer risk (health risk indices: THQ; and TTHQ) for metals/metalloid via cultured *O. mykiss* (*n* = 91) consumption in the adult population class.

Statistics	THQ_As_*	THQ_Cd_	THQ_Cr_	THQ_Cu_	THQ_Ni_	THQ_Pb_	THQ_Zn_	THQ_THg_	TTHQ
Average	0.19	0.001	0.02	0.01	0.01	0.003	0.01	0.08	0.31
SD	0.19	0.004	0.01	0.01	0.01	0.002	0.001	0.02	0.20
Reference	1.0	1.0	1.0	1.0	1.0	1.0	1.0	1.0	1.0
Non-cancer health risk	No	No	No	No	No	No	No	No	No

⁎ THQ_As_ contribution, being the highest among the trace elements analyzed, ranged from 58% to 87%.

**Table 5:** Incremental (ILCR) and cumulative (∑ILCR) lifetime cancer risks for Cr, As, Ni, and Pb through consumption of cultured *O. mykiss* (*n* = 91) in the adult population class.

	ILRC_As_	ILRC_Cr_	ILRC_Ni_	ILRC_Pb_	∑ILCR
Mean cancer risk	2.78E-01	9.82E-03	1.66E-02	2.49E-05	3.04E-01
SD	2.90E-01	3.56E-03	1.72E-02	1.87E-05	2.93E-01
Benchmark (1.0E-05)	1.00E-05	1.00E-05	1.00E-05	1.00E-05	1.00E-05
Cancer health risk	Probable	Probable	Probable	Probable	Probable

SD is the standard deviation; Exceedances in samples were ILRC_As_ (69%); ILCR_Cr_ (100%); ILCR_Ni_ (98%); ILCR_Pb_ (75%); and ∑ILCR (100%).

## 2 Experimental Design, Materials, and Methods

### 2.1 Sample collection

Ninety-one *O. mykiss* were sampled from an in-door aquaculture production system in Missouri (Fig. 1) for metal assessment. *O. mykiss* were reared from approximately 75 mm total length to harvest (age = 6 months). Culture conditions were consistent with the production of rainbow trout. Oxygen concentration and temperature measurements were taken daily while ammonia, nitrite, alkalinity, and pH were measured weekly. The feed was a complete and proprietary 42% crude protein floating feed formulated for rainbow trout and custom formulated for the hatchery that provided the fingerlings. Trout samples at maturity were collected from the rearing tanks using a hand net. The fork length and weight of samples were recorded. Additionally, 30–50 g portion of each fish was filleted, placed in polyethylene bags (Ziploc), and transported to the laboratory on ice. All samples were frozen at - 40 °C until analyses.

### 2.2 Reagents and standards

Ultrapure water (resistivity: 18.2 MΩ.cm) for the experiments was produced by a Milli-Q® Integral 5 water purification system (Millipore Corporation, USA). Reagents, and gases, standard reference material (SRM 1640a), and other quality assurance protocols were described earlier [12]. DOLT-5 (Dogfish liver certified reference material for trace metals and other constituents) and DORM-4 (Fish protein certified reference material for trace metals and other constituents) for method accuracy were acquired from the National Research Council (NRC, Ontario, Canada).

### 2.3 Microwave digestion of fish muscle samples

Microwave digestion of samples was performed in a single reaction chamber (SRC) UltraWAVE™ digestion system (maximum pressure: ∼200 bars; and temperature: 300 °C; Milestone Inc., CT, USA).  5 mL HNO_3_ was added to approximately 1.07 ± 0.03 g (ww) of fish muscle sample accurately weighed into an acid-cleaned quartz tube. Samples were digested in batches along with DORM-4 and DOLT-5 reference standards. Mineralization of samples followed the microwave heating steps: (i) 1500 W: ramp 5 min to 70 °C; (ii) 1500 W: ramp 5 min to 100 °C; (iii)) 1500 W: ramp 5 min to 180 °C; (iv) 1500 W: ramp 10 min to 250 °C; (v) 1500 W: hold at 250 °C; and (vi) cooling and depressurization. Cooled digests were quantitatively transferred into acid-cleaned 50 mL standard flasks and made up to volume with ultrapure water.  All samples including the reference standards were digested in triplicates.

### 2.4 Trace elements analysis using ICP – OES

Concentrations of seven trace elements (As, Cd, Cr, Cu, Ni, Pb, and Zn) in digested samples were quantified using an Agilent 5110 ICP-OES (synchronous vertical dual view (SVDV)). The wavelengths (nm) of measurements were Cr: 267.716; Ni: 231.604; Cd: 214.439; As: 288.812; Cu: 327.395; Pb: 220.353; and Zn: 213.857. Blanks, independent calibration verification, QCS-26 standard, and SRM 1640a solutions were analyzed along with samples. ICP Expert software (Version 7.4.1. 10449; Agilent Technologies, Inc.) calculated the analytical concentrations in samples.

The limit of detection (LOD) and limit of quantitation (LOQ) values were calculated as three times the standard deviation (3.3σ) and ten times the standard deviation (10σ) of results obtained from the analysis of 5 ppb spiked solutions. The LOD values (µg L^−1^) were Cr (4.0), As (9.0), Cd (4.0), Cu (8.0), Ni (7.0), Pb (9.0), and Zn (4.0). The LOQ values (µg/kg) were Cr (13), As (26), Cd (13), Cu (23), Ni (20), Pb (27), and Zn (12).

Recovery rates from the independent calibration verification standard solution were in the range of 103.2–105.4% and the precision ranged from 5.3 to 5.8%. For the QCS-26 standard solution, the recovery ranged from 99.3 to 102%, with precision values in the range from 0.9 to 1.7%. Regarding SRM 1640a, the recovery rates ranged from 93 to 112%, with precision values in the range from 2.0 to 11.9%. Recovery rates for DORM-4 and DOLT-5 reference samples ranged from 78 to 114% and 82–106%, respectively.

All results of elements in fish muscle tissue are expressed in mg kg^−1^ ww.

### 2.5 THg mercury analysis using DMA-80 Evo

THg concentrations in *O. mykiss* muscle samples were determined using the direct mercury analyzer (DMA-80 Evo TRICELL; Milestone Inc., USA) according to the US EPA method 7473 [4]. The method involved thermal decomposition of the sample, catalytic conversion, amalgamation, and mercury detection by atomic absorption spectrophotometry at 253.65 nm. The approximation quality for the instrument curve was > 0.996. The EasyControl software controlled the equipment operation.

LOD and LOQ were three times the standard deviation (3.3 σ) and ten times the standard deviation (10 σ) of the results of the blanks (fifteen empty quartz boats), respectively. The LOD and LOQ values were 1.5 ng/kg and 4.6 ng/kg, respectively. Certified reference materials (DORM-4: fish protein certified reference materials for trace elements; and DOLT-5: dogfish liver certified reference materials for trace metals and other constituents (NRC: National Research Council, Ottawa, ON, Canada) were used for validation and accuracy measurements. The results for DORM-4 and DOLT-5 analysis were 106 ± 2.6% and 96 ± 1.8%, respectively. The relative standard deviations for DORM- 4 and DOLT-5 were 2.52 and 1.92%, respectively. THg in samples were measured in triplicates and concentrations are reported in mg/kg ww.

The accuracy results for DORM-4 and DOLT-5 analysis were 106 ± 2.6% and 96 ± 1.8%, respectively. Likewise, the relative standard deviations for DORM- 4 and DOLT-5 were 2.52 and 1.92%, respectively.

### 2.6 Human health risk assessment

#### 2.6.1 Daily/weekly intakes (EDI/EWI)

The EDI (µg/kg body weight per day) and EWI (this study; µg/kg body weight per week) were estimated according to Eqs. (1), (2). The input variables are shown in Table 2. The EDI/EWI values (Table 3) were compared to guidelines such as those by the WHO/FAO committee.

(1) EDI=[EC*IR]/BW

(2) EWI=EDI*F

#### 2.6.2 Non-carcinogenic health risk

The health index, target hazard quotient [THQ: the ratio between exposure and the oral reference dose (RfD_o_) or provisional tolerable daily intake (PTDI) for the element] from intake of metals/metalloid by the adult population was estimated in respect to the United States Environmental Protection Agency (US EPA) method (Eq. (3)) [11].

(3) $$THQ=\frac{\left[\left(EDI\right)\left(EFr\;*\;ED\;\right)\right]}{\left[\left(\text{RfDo}\;*\;AET\right)\right]}*10^{-3}$$

All variables of Eq. (1) are shown in Table 2.

THQ > 1 signifies a significant non-carcinogenic risk to adult consumers. Conversely, THQ ≤ 1 indicates no adverse effect from non-carcinogenesis [13]. The summary results for non-carcinogenic risk in the adult risk group are reported in Table 4.

TTHQ (i.e., the sum of more than one target hazard quotient for multiple substances) was estimated in the adult population according to the US EPA method (Eq. (2)) [11].

(4) $$TTHQ=THQ_{\left(As\right)}+THQ_{\left(Pb\right)}+---+THQ_{\left(THg\right)}+THQ_{\left(Zn\right)}$$

TTHQ > 1 indicates no hazard from non-carcinogenicity in adult consumers while TTHQ ≤ 1 indicates no risk due to non-carcinogenesis [13]. The summary TTHQ values are presented in Table 4.

#### 2.6.3 Carcinogenic human health risk

The incremental lifetime cancer risk (ILCR) is the incremental probability that an individual will develop cancer during one's lifetime because of specific exposure to a carcinogenic compound [14]. The ILCR was estimated following Eqs. (5) and (6). CDI (mg/kg body weight per day) is the chronic daily intake of a chemical [11]. All variables of Eqs. (5), (6) are shown in Table 2.

(5) ILCR=CDI*CSF

(6) $$CDI=\frac{\left[EDI*EFr*ED\right]}{AET}*10^{-3}$$

The acceptable cancer risk levels for carcinogenic chemicals range from 1 × 10^−6^ (i.e., the risk of developing cancer is 1 in 1,000 000) to 1 × 10^−4^ (i.e., the risk of developing cancer is 1 in 10, 000) [14]. For the present work, the cancer risk benchmark of 10^−5^ (i.e., the risk of developing cancer is 1 in 100,000) was applied. Thus, an ILCR < 10^−6^ implies negligible cancer risk while ILCR > 10 ^−4^ signifies potential cancer risk [15]. Table 5 presents the mean cancer risks (ILCR and cumulative cancer risk (∑ILCR)) for adult consumers. The ILCR exceedances (in parenthesis) for Cr (100%), As (69%), Ni (98%), and Pb (75%) indicated probable cancer risks in the adult population. The ∑ILCR from exposure to As, Cr, Ni, and Pb in fish muscle (this study; Table 5), estimated from the individual metal/metalloid incremental cancer risk, suggested probable cancer health risk.

### 2.7 Statistical analyses

Microsoft™ Excel (Microsoft Office Professional Plus 2016) and Statgraphics Centurion 18-X64 version 17.1.04 (Statpoint Technologies, USA) were used in statistical analyses. Summary statistics of the concentration dataset performed are reported as mean ± standard deviation (SD), standard error, minimum, maximum, and percentile values. The Kolmogorov-Smirnov test of the dataset suggested non-homogeneity of variance. Following, the non-parametric Kruskal-Wallis Rank test result revealed significant statistical differences in metal/metalloid concentrations across the three fish size groups and between the genders in the large fish group. All statistical significance was accepted when *P <* 0.05.

## Ethics statement

This research project was approved by the Animal Care and Use Committee (ACUC) at the College of Agriculture, Environmental and Human Sciences, Lincoln University, Missouri (ACUC approval #: 16-2).  All animal handling and care followed the regulations and protocols of the Use of Fishes in Research (UFR) Committee.

The authors declare that they have read and follow the ethical requirements for publication in Data in Brief.

## CRediT authorship contribution statement

**Abua Ikem:** Funding acquisition, Conceptualization, Supervision, Methodology, Validation, Investigation, Formal analysis, Software, Data curation, Writing – original draft, Writing – review & editing. **Jimmie Garth:** Data curation. **James Wetzel:** Investigation, Supervision, Data curation. **Gabrielle Caldwell:** Conceptualization, Writing – review & editing.

## Declaration of Competing Interest

The authors declare that they have no known competing financial interests or personal relationships, which have or could be perceived to have influenced the work reported in this article.

## Appendix Supplementary materials

Image, application 1
